# Development and validation of an interpretable machine learning model for predicting progression-free survival after immunotherapy in patients with non-small cell lung cancer: a multicenter study

**DOI:** 10.3389/fimmu.2025.1686260

**Published:** 2025-12-19

**Authors:** Ya Li, Ji Xia, Tianchu He, Yong Hu, Daobin Zhou, Dan Zou, Benlan Li, Min Zhang, Zhongjun Huang, Mi Zhang, Xian Liu, Minfang Wang, Hongyan Luo, Fangyang Lu, Chuan Zhang, Xingxing Zhao, Shengfa Su, Jie Peng

**Affiliations:** 1 Department of Oncology, The Second Affiliated Hospital of Guizhou Medical University, Kaili, China; 2 Department of Oncology, Affiliated Hospital of Guizhou Medical University, Guiyang, China; 3 Department of Oncology, Affiliated Cancer Hospital of Guizhou Medical University, Guiyang, China; 4 Division of Oncology, School of Clinical Medicine, Guizhou Medical University, Guiyang, China; 5 Department of Oncology, Qiandongnan Prefecture People’s Hospital, Kaili, China; 6 Department of Oncology, Guiyang Pulmonary Hospital, Guiyang, China; 7 Department of Oncology, Dujiangyan Shoujia Hospital, Chengdu, China; 8 Department of Oncology, Xingyi People’s Hospital, Xingyi, China; 9 Department of Oncology, Panzhou People’s Hospital, Liupanshui, China; 10 Department of Oncology, The First People’s Hospital of Guiyang, Guiyang, China; 11 Department of Pathology, The Second Affiliated Hospital of Guizhou Medical University, Kaili, China

**Keywords:** NSCLC, immunotherapy, CtDNA, PFS, XGBoost

## Abstract

**Background:**

This study aimed to develop and validate an interpretable machine learning model that harnesses circulating tumor DNA (ctDNA) to predict progression-free survival (PFS) in patients with non-small cell lung cancer (NSCLC) undergoing immunotherapy, thereby addressing the inherent limitations of conventional biomarkers such as PD-L1 expression and tumor mutational burden.

**Methods:**

This multicenter study involved pretreatment ctDNA profiling of 441 patients with non-small cell lung cancer (NSCLC), stratified into three independent cohorts: a training set (n=303, OAK trial), a validation set (n=97, POPLAR trial), and a local test set (n=41, multicenter retrospective cohort, 2023–2024). Using 5-fold cross-validated LASSO-Cox (Least Absolute Shrinkage and Selection Operator-Cox Proportional Hazards) regression, 25 prognostic genomic features were identified for integration into an eXtreme Gradient Boosting (XGBoost) model. Model performance was systematically evaluated via three approaches: (1) discrimination metrics, including AUC with 95% confidence intervals, accuracy, sensitivity, and specificity; (2) Kaplan-Meier survival analysis complemented by log-rank testing; and (3) SHapley Additive exPlanations (SHAP) for interpreting feature importance.

**Results:**

The model exhibited robust predictive performance, with AUCs of 0.82 (training cohort), 0.79 (validation cohort), and 0.77 (test cohort). Key genomic predictors included TP53 mutations, which were associated with shorter PFS, and BRCA2 mutations, which correlated with longer PFS. SHAP analysis identified NOTCH1 as a novel predictive biomarker, whose feature contribution profile suggests a role in immune modulation in lung squamous cell carcinoma. Risk stratification significantly distinguished PFS outcomes (log-rank *P* < 0.05). Decision curve analysis confirmed the model’s clinical utility, as it outperformed “treat-all” strategies.

**Conclusion:**

This study establishes a robust, interpretable ctDNA-derived machine learning algorithm for predicting PFS in NSCLC patients receiving immune checkpoint inhibitors. The identification of TP53, BRCA2, and NOTCH1 as biologically plausible predictive biomarkers advances understanding of immunotherapy response mechanisms and enables clinically actionable risk stratification to guide therapeutic decision-making. These findings underscore the need for prospective multicenter validation to facilitate translation into precision oncology practice.

## 1 Introduction

According to the latest statistics, lung cancer remains the leading cause of cancer morbidity and mortality worldwide, topping the list of cancer-related deaths for ten consecutive years (1). Histopathologically, lung cancer is categorized into two main types: non-small cell lung cancer (NSCLC), which accounts for approximately 80% of cases and includes adenocarcinoma, squamous cell carcinoma, and other subtypes; and small cell lung cancer (2, 3). Recent advances in immunotherapy have conferred substantial clinical benefits to an expanding cohort of NSCLC patients (4). Immune checkpoint inhibitors targeting programmed cell death protein 1 (PD-1) and programmed death-ligand 1 (PD-L1) have emerged as a cornerstone of treatment for advanced NSCLC (5). By modulating immune inhibitory pathways, these agents enhance the immune system’s capacity to recognize and eliminate tumor cells, thereby controlling tumor progression and metastasis (6). Clinical evidence confirms that immune checkpoint inhibitors significantly improve survival outcomes in patients with NSCLC (7–10).

While ICIs have become a cornerstone treatment, they represent one facet of a broader movement towards precision oncology. This paradigm is further exemplified by the development of sophisticated nanomaterial-based therapies, which seek to achieve spatiotemporal control over treatment. Examples include light-activated nanopolyplexes for targeted gene silencing (11), multifunctional graphene derivatives for integrated diagnosis and therapy (12), and gold nanorod-based platforms that synergize photothermal ablation with immunotherapy (13). A fundamental challenge unifying all these advanced modalities, however, is the reliable identification of patients who will respond.

However, clinical responses to immunotherapy are highly heterogeneous, with only a subset of patients deriving meaningful benefit (14). Currently, biomarkers such as PD-L1 expression levels and tumor mutational burden (TMB) are limited by suboptimal sensitivity and detection accuracy, highlighting the need for more precise monitoring tools to optimize treatment decisions (15). Circulating tumor DNA (ctDNA), a core component of liquid biopsies, provides real-time insights into tumor genomic profiles and disease burden, facilitating more accurate prediction of immunotherapeutic efficacy (16). Progression-free survival (PFS) is a key metric for evaluating the clinical benefits of immunotherapy (17). Given that tumor patients often experience pseudoprogression following immunotherapy (18, 19), this study selected PFS as the primary endpoint rather than short-term efficacy measures (such as objective response rate). Moreover, dynamic changes in ctDNA have been shown to closely correlate with tumor treatment response and disease progression (20). Compared with traditional tissue biopsies, ctDNA testing is minimally invasive, readily repeatable, and capable of sensitively monitoring tumor clonal evolution and minimal residual disease. Multiple studies have demonstrated that machine learning algorithms can effectively predict the short-term efficacy of immunotherapy in cancer patients (21–25). Nevertheless, reliable ctDNA-based PFS prediction models remain elusive.

This study aimed to develop and validate a machine learning model for predicting PFS in NSCLC patients using pre-immunotherapy ctDNA data. After evaluating multiple machine learning algorithms—including random forest, logistic regression, support vector machines, and eXtreme Gradient Boosting (XGBoost)—we selected XGBoost for its superior performance in processing high-dimensional genomic data, robustness against overfitting, and model interpretability; these features are critical for clinical translation.

## 2 Methods

This study analyzed ctDNA data from two clinical trials: the OAK trial (a phase III, open-label, multicenter randomized controlled trial comparing atezolizumab with docetaxel in previously treated NSCLC patients) and the POPLAR trial (a phase II, open-label, multicenter randomized controlled trial evaluating atezolizumab versus docetaxel in advanced NSCLC). We included data from 425 patients in the OAK trial and 144 patients in the POPLAR trial who received immunotherapy. Additionally, we collected ctDNA data from 52 NSCLC patients treated at the Second Affiliated Hospital of Guizhou Medical University, People’s Hospital of Qiandongnan Region, and Guiyang Pulmonary Hospital between January 2023 and June 2024. The study was approved by the Ethics Committee of The Second Affiliated Hospital of Guizhou Medical University (Approval No. 2023-LS-02). As a retrospective study, this research strictly adhered to the Declaration of Helsinki (26), with full confidentiality of patient information ensured; a waiver of informed consent was granted by the institutional review board due to the retrospective nature of the analysis.

**Figure 1:**
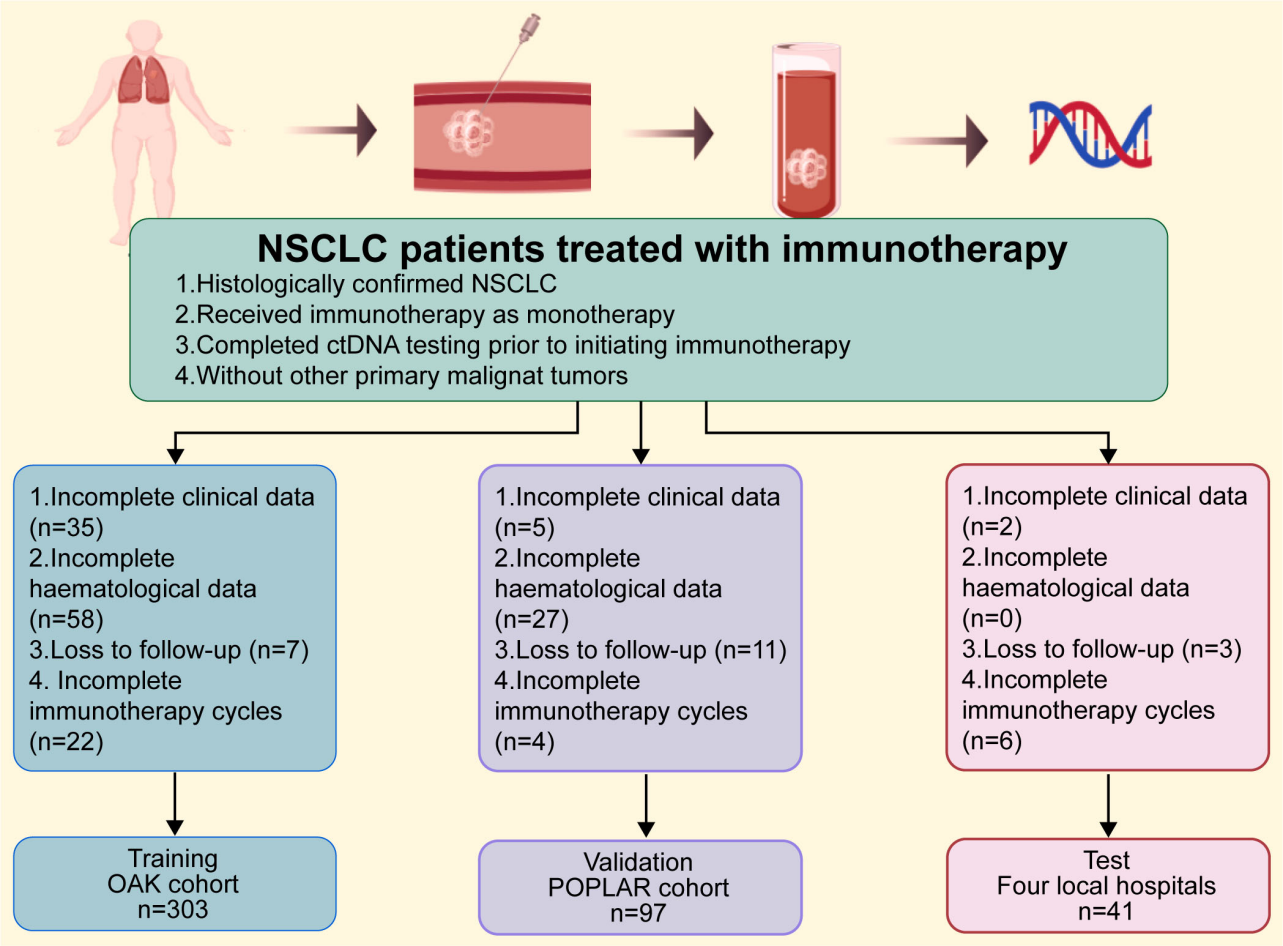
Experimental flowchart.

**Figure 1** displays the flow chart. Inclusion criteria: (a) Histologically confirmed NSCLC; (b) Received immunotherapy as monotherapy; (c) Completed ctDNA testing prior to initiating immunotherapy; (d) Without other primary malignant tumors. Exclusion criteria: (a) Incomplete clinical data; (b) Incomplete hematological data; (c) Lost to follow-up; (d) Incomplete immunotherapy course. The primary endpoint was PFS within 2 years, defined as the time from treatment initiation to disease progression or death from any cause. Data from the OAK trial served as the training set (n=303), data from the POPLAR trial as the validation set (n=97), and data from the three local hospitals as the test set (n=41). The training (OAK) and validation (POPLAR) datasets were obtained as analysis-ready datasets from www.clinicalstudydatarequest.com. No missing values were present in the acquired datasets; hence, no imputation was performed. The genomic features were binary variables (mutated: 1, wild-type: 0), and as such, no normalization or feature transformation was applied. The same preprocessing logic was applied to the internal test set. The full analysis code is available at (https://github.com/Yali15207856138/improved-sn).

After cohort assignment, we analyzed baseline characteristics (age, gender, histological type, and TMB expression level) across the three groups and generated a table of baseline characteristics (**Table 1**) using SPSS software (v26.0). For the training set, univariate Cox regression analysis was performed on ctDNA data. Genes with *P* < 0.2 in the univariate analysis were included in multivariate Cox regression, with those exhibiting *P* < 0.05 retained (**Supplementary Table 1**). Following gene selection via 5-fold cross-validated LASSO-Cox regression (glmnet package in R v3.5.1), the selected features were incorporated into an XGBoost model (Python v3.0.1). Prior to final model building, the XGBoost hyperparameters were optimized through an iterative process guided by 5-fold cross-validation performance (AUC). We explored a broad parameter grid, including but not limited to: learning_rate [0.01, 0.05, 0.1, 0.15, 0.2], max_depth [3, 4, 5, 6, 7], subsample [0.7, 0.8, 0.9, 1.0], and reg_lambda [0.1, 0.5, 1.0, 1.5, 2.0]. The final parameter set was selected as it yielded the highest and most stable cross-validated AUC, configured as follows: booster = ‘gbtree’, objective = ‘binary:logistic’, n_estimators = 100, learning_rate = 0.10, max_depth = 6, subsample = 0.80, reg_lambda = 1.00, with gamma = 0.00 and reg_alpha = 0.00. The model output yielded prediction probabilities, which were used to calculate AUC, accuracy, sensitivity, specificity, positive predictive value (PPV), and negative predictive value (NPV).

**Table 1:** Baseline table.

Variables	Total (n = 441)	Training set (n = 303)	Validation set (n = 97)	Test set (n=41)	*P*
Gender, n(%)					0.113
Female	156 (35.37)	115 (37.95)	32 (32.99)	9 (21.95)	
Male	285 (64.63)	188 (62.05)	65 (67.01)	32 (78.05)	
Age, n(%)					0.111
<50	33 (7.48)	18 (5.94)	12 (12.37)	3 (7.32)	
≥50	408 (92.52)	285 (94.06)	85 (87.63)	38 (92.68)	
Histology, n(%)					<0.001
Squamous cell carcinoma	142 (32.20)	80 (26.40)	34 (35.05)	28 (68.29)	
Non-squamous cell carcinoma	299 (67.80)	223 (73.60)	63 (64.95)	13 (31.71)	
TMB, n(%)					0.256
<10	265 (60.09)	182 (60.07)	54 (55.67)	29 (70.73)	
≥10	176 (39.91)	121 (39.93)	43 (44.33)	12 (29.27)	

Kaplan-Meier survival analysis was performed using the survival package in R, generating PFS rate estimates and survival curves. Patients were stratified into risk groups based on the optimal cutoff value, which was determined using the Youden index. Survival differences between groups were assessed using the log-rank test. To evaluate model generalizability, calibration curves were generated and decision curve analysis (DCA) was conducted using R. To interpret model behavior, we applied SHapley Additive exPlanations (SHAP) analysis (R v3.5.1), which included SHAP feature importance analysis to identify key predictive features and SHAP force plots to visualize individual prediction mechanisms (27). This integrated approach reveals both global feature contributions and instance-level decision patterns. Statistical significance was defined as P < 0.05 for all hypothesis tests.

## 3 Results

A total of 441 patients were included in this study. **Table 1** summarizes their clinical characteristics. No significant differences in the distribution of gender, age, and TMB expression level were observed in the training set, validation set, and local test set. However, histological type distributions differed: squamous cell carcinoma comprised 68.29% of the local test set (*vs*. 31.71% non-squamous), whereas the opposite trend was noted in the training and validation sets.

**Figure 2:**
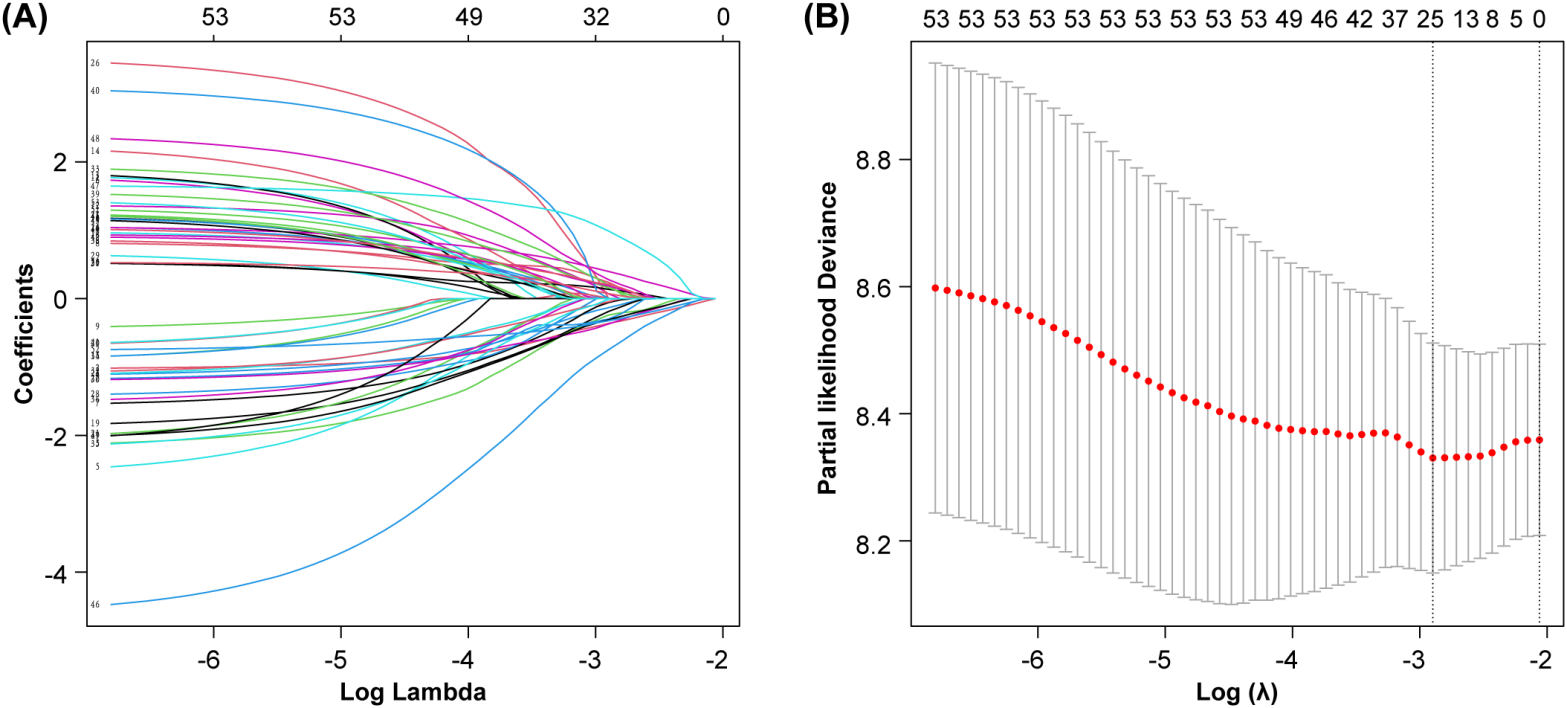
Feature selection by Lasso-Cox regression. **(A)** Coefficient curves. **(B)** Cross-validation curve.

**Figure 3:**
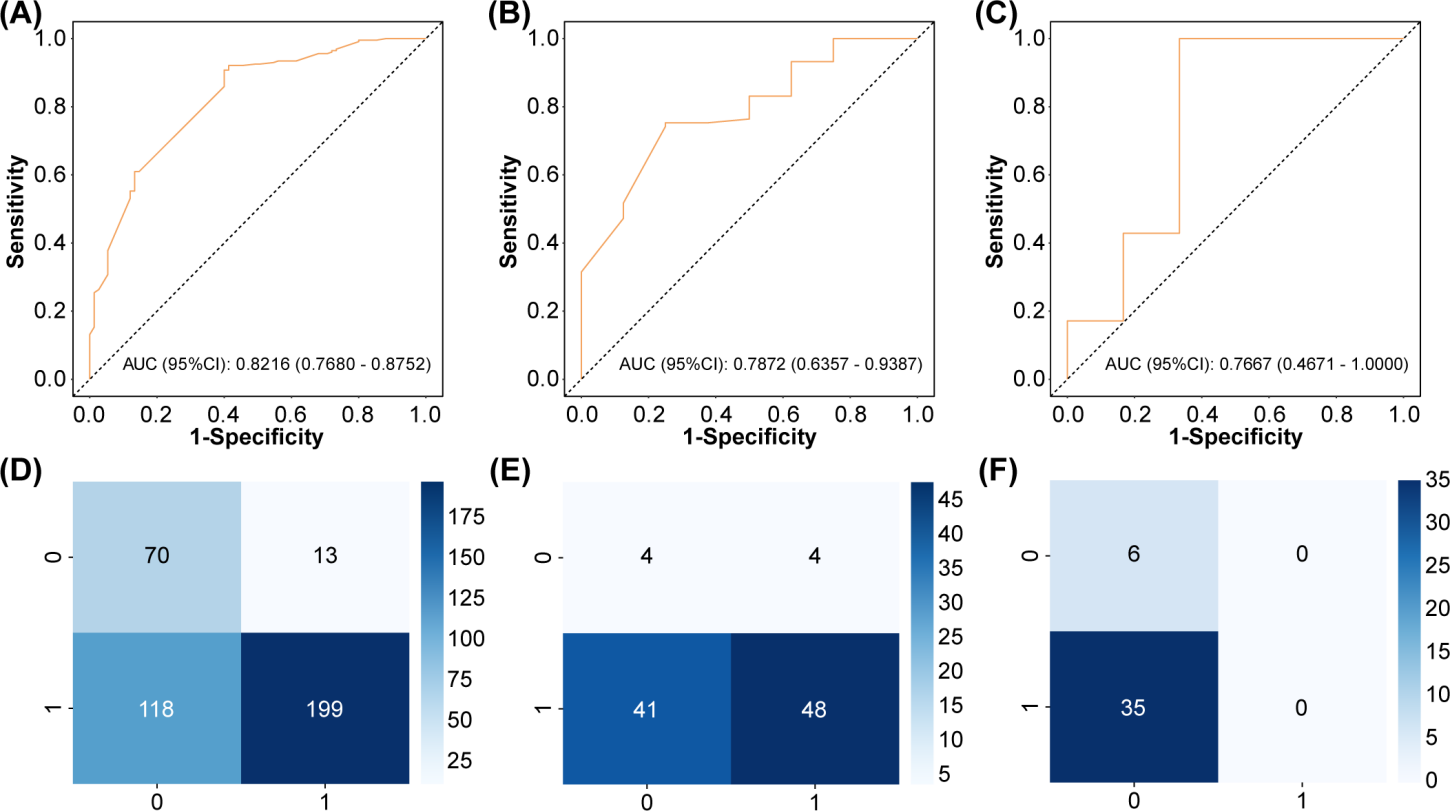
Model performance evaluation across datasets. **(A)** Training set ROC curve. **(B)** Validation set ROC curve. **(C)** Test set ROC curve. **(D)** Training set confusion matrix. **(E)** Validation set confusion matrix. **(F)** Test set confusion matrix.

From the 192 initial gene features extracted from 441 patients, 5-fold cross-validated LASSO-Cox regression identified 25 significant gene features (**Figure 2**). As shown in **Table 2** and **Figure 3**, the training set yielded an AUC of 0.82 (95% CI: 0.77–0.88), while the validation and local test sets demonstrated AUCs of 0.79 (95% CI: 0.64–0.94) and 0.77 (95% CI: 0.47–1.00), respectively. The confusion matrix (**Figure 3**) and performance metrics (**Table 2**) confirmed consistent model performance across the training set (n=303), validation set (n=97), and independent test set (n=41), with accuracies of 83% (95% CI: 78–87%), 75% (65–83%), and 95% (83–99%), respectively. Sensitivity values were 60%, 75%, and 67%, while specificity reached 91%, 75%, and 100% across the three datasets. This relatively stable performance indicates no significant overfitting, although the higher accuracy observed in the small test set necessitates further validation in larger cohorts. In addition, we developed an integrated model combining the 25 screened gene features with clinical data (gender, age, pathology type, and TMB expression level). However, the prediction performance did not show any significant improvement. The corresponding ROC curves for the validation and local test sets are presented in **Supplementary Figure 1**.

**Table 2:** Model evaluation table.

Statistical value	Training set	Validation set	Test set
Accuracy	0.83 (0.78-0.87)	0.75 (0.65-0.83)	0.95 (0.83-0.99)
Sensitivity (95%CI)	0.60 (0.49 - 0.71)	0.75 (0.45 - 1.00)	0.67 (0.29 - 1.00)
Specificity (95%CI)	0.91 (0.87 - 0.95)	0.75 (0.66 - 0.84)	1.00 (1.00 - 1.00)
PPV (95%CI)	0.68 (0.57 - 0.79)	0.21 (0.06 - 0.37)	1.00 (1.00 - 1.00)
NPV (95%CI)	0.87 (0.83 - 0.92)	0.97 (0.93 - 1.00)	0.95 (0.87 - 1.00)
AUC (95%CI)	0.82 (0.77-0.88)	0.79 (0.64-0.94)	0.77 (0.47-1.00)

PPV, Positive Predictive Value; NPV, Negative Predictive Value; AUC, area under the curve.

**Figure 4:**
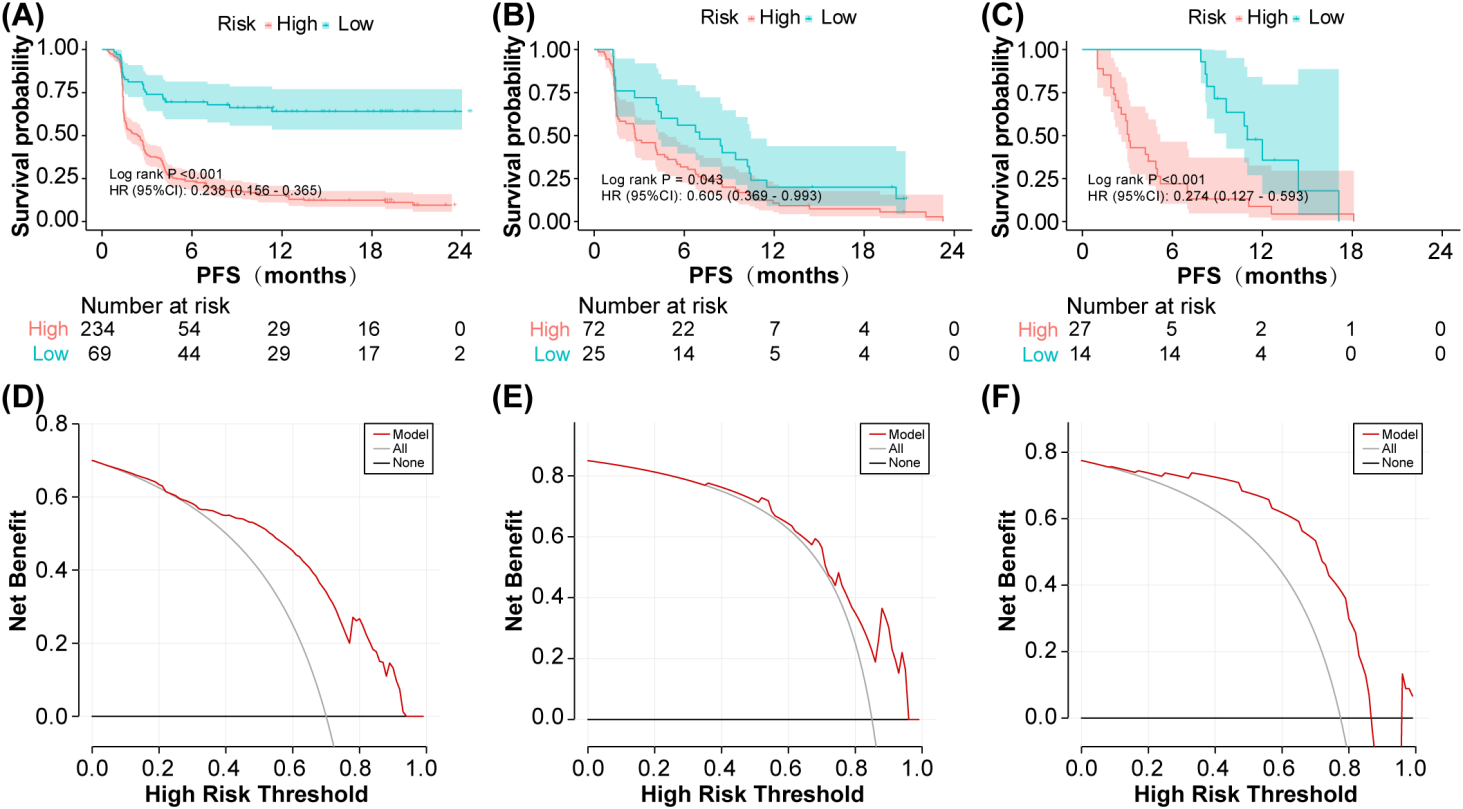
Validation of progression-free survival prediction and clinical utility. **(A)** Training set Kaplan-Meier curve for PFS. **(B)** Validation set Kaplan-Meier curve for PFS. **(C)** Test set Kaplan-Meier curve for PFS. **(D)** Training set decision curve analysis. **(E)** Validation set decision curve analysis. **(F)** Test set decision curve analysis.

The optimal model threshold was determined by maximizing the Youden index (0.653). Risk stratification using the model revealed significant differences in tumor progression rates (log-rank test *P* < 0.001 for the training set, *P* = 0.043 for the validation set, and *P* < 0.001 for the local test set; **Figure 4**). Given the small sample sizes of the validation and local test sets, we further performed decision curve analysis (DCA) and generated calibration plots to assess the model’s clinical utility. As shown in the DCA (**Figure 4**) and calibration plot (**Supplementary Figure 2**), the model’s predictions strongly correlated with patients’ actual tumor progression status.

**Figure 5:**
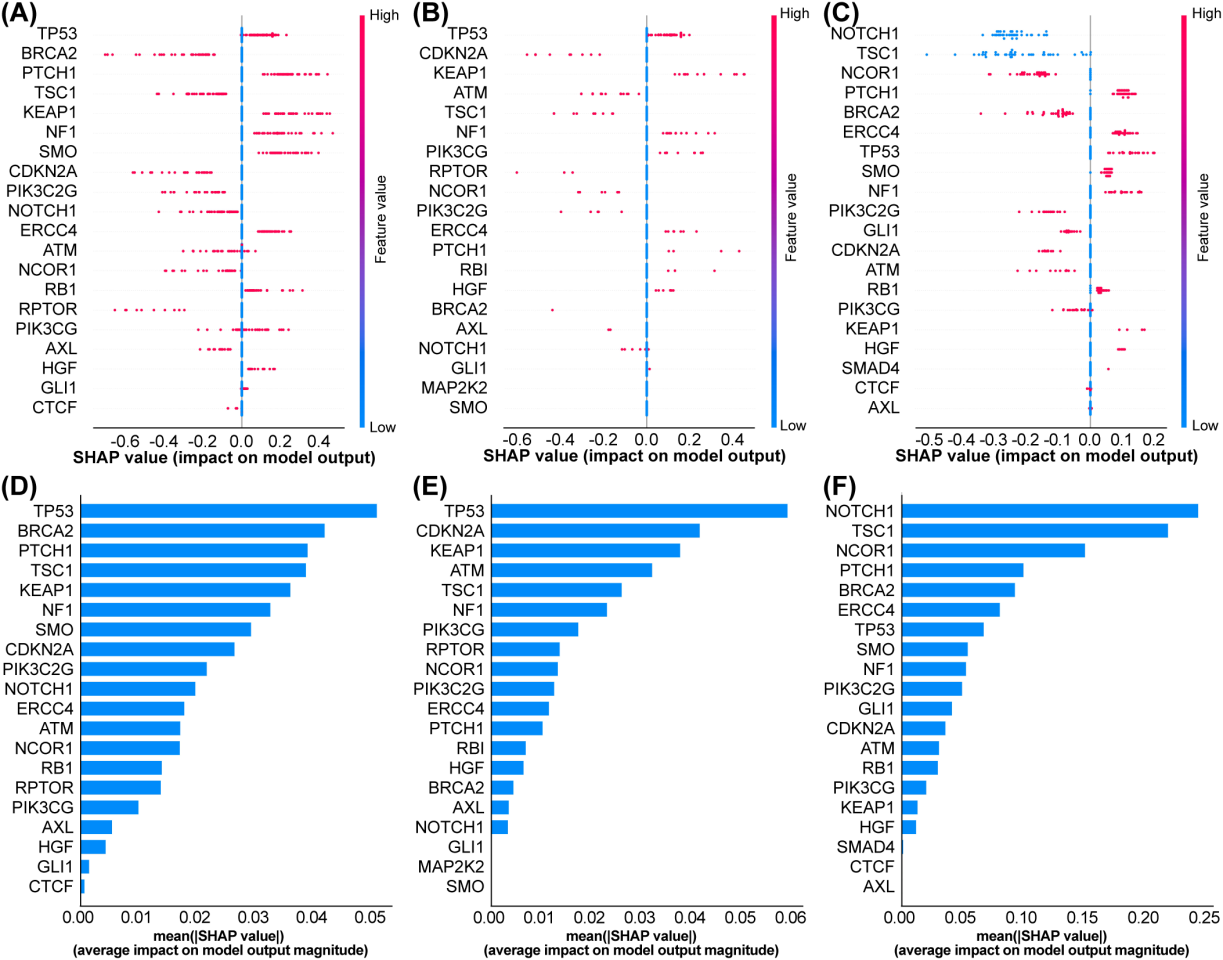
Consistent interpretation of feature importance via SHAP analysis across datasets. **(A)** Training set SHAP feature contribution plot. **(B)** Validation set SHAP feature contribution plot. **(C)** Test set SHAP feature contribution plot. **(D)** Training set SHAP importance plot. **(E)** Validation set SHAP importance plot. **(F)** Test set SHAP importance plot.

**Figure 6:**
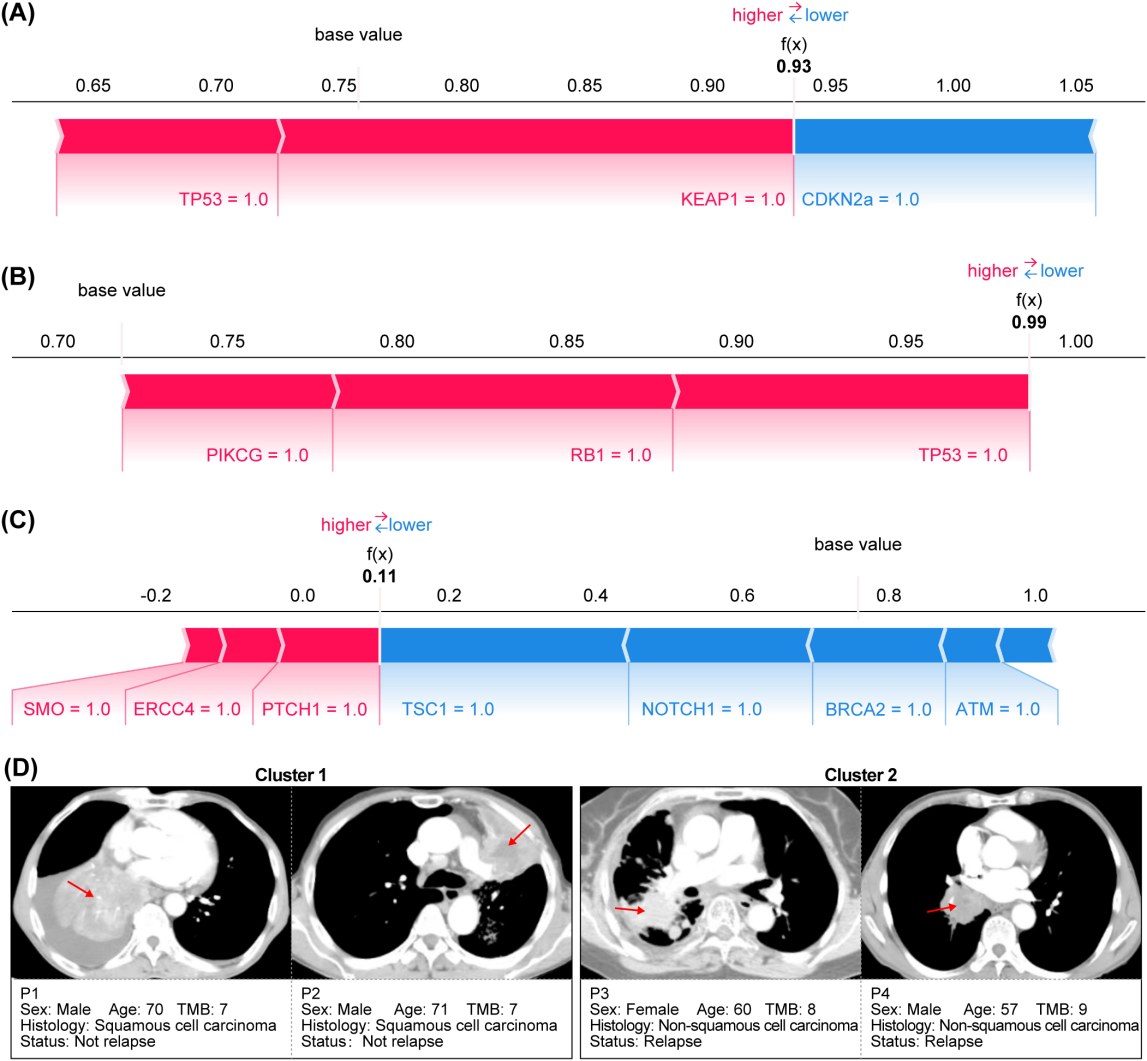
Model interpretability analysis with clinical exemplars. **(A)** Training set SHAP sample plot. **(B)** Validation set SHAP sample plot. **(C)** Test set SHAP sample plot. **(D)** Representative CT images of test set patients stratified by treatment response: two non-progressors (top) and two progressors (bottom).

In the training set, TP53, BRCA2, and PTCH1 exhibited the highest absolute average SHAP values (**Figure 5**), indicating that their mutation statuses contributed most to the model’s predictions of poor outcomes. Moreover, across all three datasets, TP53 mutations were consistently associated with shorter PFS (reflected by negative SHAP values, indicating higher risk), whereas BRCA2 mutations correlated with longer PFS (positive SHAP values, indicating a protective effect against disease progression). **Figure 6** shows the SHAP values for a representative single sample in the training set (A), validation set (B), and test set (C), as well as CT images of four representative patients illustrating the two treatment outcomes in the test set (D). Consistent patterns were observed across all datasets: TP53 mutations (red bars) positively contributed to higher risk predictions, while BRCA2 mutations (blue bars) exerted protective effects. This consistency underscores the robustness of the model’s feature interpretation.

## 4 Discussion

The model’s ability to maintain consistent performance across distinct datasets (training AUC: 0.82; validation AUC: 0.79; test AUC: 0.77) is particularly notable, considering the histological heterogeneity among cohorts. This robustness suggests that the identified genomic signatures reflect fundamental biological processes rather than dataset-specific artifacts. The significant inverse association between TP53 mutations and PFS aligns with accumulating evidence that TP53 dysfunction fosters an immunosuppressive tumor microenvironment (28–30). Recent studies demonstrate that TP53 mutations can downregulate antigen presentation machinery (e.g., MHC class I) and recruit myeloid-derived suppressor cells, collectively promoting an immune-evasive phenotype (31–33). This mechanistic plausibility strengthens the biological validity of our model’s predictions. Our findings contribute to a growing body of evidence that diverse molecular alterations converge on an immunosuppressive TME. Beyond the genetic mutations captured here, epigenetic dysregulation represents a parallel pathway to immune evasion. For instance, in mucosal melanoma, hypomethylation-driven overexpression of CNDP1 was recently linked to a ‘cold’ TME and inferior immunotherapy outcomes (34). More directly relevant to NSCLC, Yuan et al. demonstrated that the histone methyltransferase KMT5C promotes immune evasion by activating DNA damage repair to suppress the STING-IRF3-type I interferon pathway, thereby inhibiting CD8+ T cell recruitment and function. Crucially, they showed that high KMT5C expression predicts poor response to immune checkpoint blockade (35). These studies collectively underscore that both the genomic features identified by our model and broader epigenetic mechanisms can shape a TME refractory to immunotherapy.

The protective effect associated with BRCA2 mutations represents a complex yet compelling finding. Although initially counterintuitive, the correlation between BRCA2 mutations and longer PFS in our model is consistent with two potential mechanisms: (i) adaptive silencing of the spindle assembly checkpoint (via NSFL1C/AURKB downregulation) to mitigate genomic instability, as reported in BRCA2-deficient prostate cancer (36), and (ii) enhanced tumor immunogenicity stemming from accumulated DNA damage. These dual vulnerabilities may collectively increase the susceptibility of BRCA2-mutated tumors to immunotherapy. Our results complement recent work by Samstein et al., who reported improved outcomes in BRCA2-mutated tumors across multiple cancer types treated with immunotherapy (37). The consistency of this association across all three datasets not only reinforces its validity but also positions BRCA2 status as a candidate predictive biomarker for immunotherapy response, warranting prospective validation.

In the test set feature importance analysis, NOTCH1 emerged unexpectedly as a prominent predictor, warranting further discussion. While direct evidence linking NOTCH1 to PD-L1 regulation in squamous cell carcinoma remains limited – and the relationship is complex and context-dependent – research indicates that NOTCH1 plays a multifaceted role in this cancer type (38). Activation of NOTCH1 signaling pathway can function as an oncogenic driver, potentially promoting PD-L1 expression indirectly to establish an immunosuppressive microenvironment (39). Conversely, loss-of-function mutations in NOTCH1 (where it acts as a tumor suppressor) may lead to hyperactivation of the NF-κB signaling pathway, resulting in upregulated PD-L1 expression (40, 41). This represents an indirect regulatory mechanism mediated through the release of NF-κB inhibition, previously reported in head and neck squamous cell carcinoma. Furthermore, NOTCH1 signaling frequently cross talks with pathways such as RAS/MAPK and PI3K/AKT – established regulators of PD-L1 expression (42). Thus, it is plausible that NOTCH1 indirectly modulates PD-L1 expression in lung squamous cell carcinoma through these or related mechanisms, though this requires confirmation in future studies. Given the overrepresentation of squamous cell carcinoma in our test set (68.29%), the prominence of NOTCH1 in our analysis may reflect squamous cell carcinoma-specific biological interactions or dependencies involving this gene, which merit further investigation.

While PD-L1 expression remains the gold standard for immunotherapy selection, our model’s AUC (0.77–0.82) compares favorably with the predictive accuracy of PD-L1, which typically ranges from 0.65 to 0.70 in meta-analyses (15). This improvement likely stems from capturing multidimensional genomic information beyond a single immune checkpoint marker. Notably, our approach diverges from TMB-based methods by prioritizing functional mutations over total mutation burden. This distinction may explain why we detected predictive signals from BRCA2—a low-frequency mutation—often missed by TMB-centric approaches (37). The predictive power of our model, which integrates signals from multiple genes, finds strong independent validation in the recent work on KMT5C. The power of a multi-gene approach to capture such complex biology is further exemplified by recent research beyond NSCLC. For instance, a very recent study by Liu developed and validated a prognostic model based on circadian rhythm-related genes (CRGs) in skin cutaneous melanoma (43). Despite the different cancer type and gene set, their model similarly identified distinct immune subtypes and demonstrated a strong association between a high-risk CRG score and an immunosuppressive TME, characterized by upregulated immune checkpoints and reduced sensitivity to therapy. The convergence of findings—that multi-gene signatures derived from disparate biological contexts (somatic mutations in NSCLC *vs*. circadian genes in SKCM) consistently predict TME status and therapeutic outcome—considerably strengthens the validity and generalizability of the integrative genomics approach that underpins our model. The fact that a single epigenetic regulator like KMT5C can profoundly influence the TME and immunotherapy response underscores the biological rationale for why multi-gene models like ours are necessary to capture the complex determinants of treatment outcome (35). While recent studies have explored ctDNA for early relapse detection or resistance monitoring (44–47), our work uniquely focuses on baseline genomic predictors of PFS, providing a clinically actionable tool for risk stratification prior to treatment initiation.

Our work addresses two critical gaps in translational machine learning: reproducibility and interpretability. By leveraging SHAP values, we transcend “black box” predictions to identify biologically plausible drivers of model behavior. For instance, the directional consistency of TP53 and BRCA2 effects across the training and validation phases (**Figures 5**, **6**) confirms these are genuine biological signals rather than overfitting artifacts. Decision curve analysis (**Figure 4**) further substantiates the model’s clinical utility. At a threshold probability of 30%—reflecting real-world clinical willingness to intervene—the model demonstrated a superior net benefit compared to both “treat-all” and “treat-none” strategies. This suggests potential cost savings by avoiding ineffective therapies in predicted non-responders, a critical consideration given the substantial economic burden of immunotherapy (48). From a translational standpoint, our findings carry several immediate implications (1): Risk stratification: The model can identify high-risk patients—particularly those with TP53 mutations—who may derive greater benefit from more aggressive or combination therapeutic strategies (2). Treatment selection: BRCA2-mutated patients might represent ideal candidates for immunotherapy monotherapy (3). Trial design: The model could function as an enrichment tool in future clinical trials. Notably, the excellent negative predictive value (95% in the test set) suggests particular utility in avoiding unnecessary treatment for low-risk patients. This addresses a critical current challenge in NSCLC immunotherapy: overtreatment, wherein a substantial proportion of patients experience toxicity without clinical benefit.

The model’s performance is particularly noteworthy given the challenges posed by the test set’s distinct histological composition (68.29% squamous cell carcinoma *vs*. 26.40% in the training set). This suggests the identified genomic signatures may transcend histological subtypes, potentially reflecting fundamental mechanisms of tumor-immune interaction. The strategic use of LASSO-Cox regression for feature selection prior to XGBoost modeling proved particularly effective, as evidenced by the model’s ability to identify clinically relevant genes such as NOTCH1—ones that might have been overlooked by conventional approaches.

We also observed that when ctDNA was combined with clinical data (gender, age, histologic type, TMB expression level), the validation set (AUC: 0.787 *vs*. 0.770) and the test set (AUC: 0.767 *vs*. 0.686) performed worse than the ctDNA-only model. This counterintuitive phenomenon may stem from three reasons: First, baseline characterization revealed significant histological distribution bias in the test set (68.29% squamous carcinoma), where clinical factors are highly sensitive to distributional variations, potentially reducing the generalizability of the combined model. Second, the 25 ctDNA features capture molecular heterogeneity of tumors, suggesting their biological information may overshadow the predictive value of clinical phenotypes (e.g., the NOTCH1 gene). Third, the limited sample size of the test set (n=41) combined with increased model complexity amplified overfitting risk, implying that pure ctDNA-based models may offer greater robustness than traditional clinical-ctDNA integration in precision medicine. This highlights the “more features ≠ better performance” paradox in biomarker research, emphasizing that biological relevance outweighs feature quantity (49).

While our results are promising, several limitations warrant consideration (1): The retrospective design introduces potential biases in patient selection (2). The relatively small test set (n=41) constrains the precision of performance estimates (3). The model does not currently incorporate imaging information, such as CT and MRI. Moving forward, we plan to conduct prospective validation in larger multicenter cohorts, further integrate radiological features and serial ctDNA measurements, and perform deeper mechanistic studies to clarify the biological basis underlying NOTCH1’s predictive role.

## 5 Conclusions

This study developed an interpretable ctDNA-based machine learning model for predicting PFS in NSCLC patients receiving immunotherapy. SHAP analysis identified TP53, BRCA2, and other genomic predictors, while elucidating their underlying biological mechanisms. The model’s consistent performance across diverse datasets highlights its clinical potential, although prospective validation is required to guide personalized therapeutic strategies.

## Data Availability

The datasets presented in this study can be found in online repositories. The names of the repository/repositories and accession number(s) can be found below: https://github.com/Yali15207856138/improved-sn, www.clinicalstudydatarequest.com.

## References

[1] Rebecca LS Angela NG Ahmedin J . Cancer statistics, 2024. CA: Cancer J Clin. (2024). doi: 10.3322/caac.21820, PMID: 38230766

[2] Nicolas G . New strategies and novel combinations in EGFR TKI-resistant non-small cell lung cancer. Curr Treat options Oncol. (2022). doi: 10.1007/s11864-022-01022-7, PMID: 36242712

[3] Larissa AP Varune Rohan R Stephen L Wan LL . Genetic alterations defining NSCLC subtypes and their therapeutic implications. Lung Cancer (Amsterdam Netherlands). (2013). doi: 10.1016/j.lungcan.2013.07.025, PMID: 24011633

[4] Aritraa L Avik M Pravin DP Navneet S Purvish MP Bharti B . Lung cancer immunotherapy: progress, pitfalls, and promises. Mol Cancer. (2023). doi: 10.1186/s12943-023-01740-y, PMID: 36810079 PMC9942077

[5] Michael JG Roy SH Sarah BG . Selecting the optimal immunotherapy regimen in driver-negative metastatic NSCLC. Nat Rev Clin Oncol. (2021). doi: 10.1038/s41571-021-00520-1, PMID: 34168333

[6] Julian AMA ErinMarie OK Yanyan L . Next generation of immune checkpoint inhibitors and beyond. J Hematol Oncol. (2021). doi: 10.1186/s13045-021-01056-8, PMID: 33741032 PMC7977302

[7] Leena G Delvys RA Shirish MG Emilio E Enriqueta F Flávia De A . Pembrolizumab plus chemotherapy in metastatic non-small-cell lung cancer. New Engl J Med. (2018). doi: 10.1056/NEJMoa1801005, PMID: 29658856

[8] Silvia N Dariusz MK Alexander L Mahmut G David V Julien M . Pembrolizumab plus chemotherapy in squamous non-small-cell lung cancer: 5-year update of the phase III KEYNOTE-407 study. J Clin Oncol Off J Am Soc Clin Oncol. (2023). doi: 10.1200/JCO.22.01990, PMID: 36735893 PMC10082300

[9] Heather AW Moïshe L Terufumi K Masahiro T Se-Hoon L Shugeng G . Perioperative pembrolizumab for early-stage non-small-cell lung cancer. New Engl J Med. (2023). doi: 10.1056/NEJMoa2302983, PMID: 37272513 PMC11074923

[10] Marina Chiara G Shirish MG Giovanna S Enriqueta F Emilio E Manuel D . Pembrolizumab plus pemetrexed and platinum in nonsquamous non-small-cell lung cancer: 5-year outcomes from the phase 3 KEYNOTE-189 study. J Clin Oncol Off J Am Soc Clin Oncol. (2023). doi: 10.1200/JCO.22.01989, PMID: 36809080 PMC10082311

[11] Jingchao J Jing Y Leimin Q Biao Z Xiaodong T Shuanghai L . Controlled siRNA release of nanopolyplex for effective targeted anticancer therapy in animal model. Int J nanomedicine. (2024). doi: 10.2147/IJN.S443636, PMID: 38344438 PMC10859097

[12] Li Wen C Lu Yao F Zhiyong S . Application research progress of nanomaterial graphene and its derivative complexes in tumor diagnosis and therapy. Curr medicinal Chem. (2024). doi: 10.2174/0109298673251648231106112354, PMID: 38299292

[13] Cao K Zhou Y Shen Y Wang Y Huang H Zhu H . Combined photothermal therapy and cancer immunotherapy by immunogenic hollow mesoporous silicon-shelled gold nanorods. J Pharm Sci. (2024) 113:2232–44. doi: 10.1016/j.xphs.2024.03.007, PMID: 38492845

[14] Xin Y Chaonan H Chunxia S . Immunotherapy resistance of lung cancer. Cancer Drug resistance (Alhambra Calif). (2022). doi: 10.20517/cdr.2021.101, PMID: 35582531 PMC8992581

[15] Steve L Julie ES David LR Daphne WW Michael AB Douglas BJ . Comparison of biomarker modalities for predicting response to PD-1/PD-L1 checkpoint blockade: A systematic review and meta-analysis. JAMA Oncol. (2019). doi: 10.1001/jamaoncol.2019.1549, PMID: 31318407 PMC6646995

[16] Jiachen X Rui W Yiran C Shangli C Lin W Baolan L . Circulating tumor DNA-based stratification strategy for chemotherapy plus PD-1 inhibitor in advanced non-small-cell lung cancer. Cancer Cell. (2024). doi: 10.1016/j.ccell.2024.08.013, PMID: 39255777

[17] Yunfang Y Dongqiang Z Qiyun O Shengbo L Anlin L Yongjian C . Association of survival and immune-related biomarkers with immunotherapy in patients with non-small cell lung cancer: A meta-analysis and individual patient-level analysis. JAMA network Open. (2019). doi: 10.1001/jamanetworkopen.2019.6879, PMID: 31290993 PMC6625073

[18] Marina AL Denis SF Pardabekova OA Nosova MV Veronika T Vladimir L . The efficacy of immune checkpoint inhibitors in patients with cancer with pseudoprogression. J Clin Oncol. (2024). doi: 10.1200/jco.2024.42.16_suppl.2620

[19] Meng-Yu C Yue-Can Z . Pseudoprogression in lung cancer patients treated with immunotherapy. Crit Rev oncology/hematology. (2021). doi: 10.1016/j.critrevonc.2021.103531, PMID: 34800651

[20] Xinghao A Bo J Zhiyi H Junping Z Minglei Z Jun Z . Noninvasive early identification of durable clinical benefit from immune checkpoint inhibition: a prospective multicenter study (NCT04566432). Signal transduction targeted Ther. (2024). doi: 10.1038/s41392-024-02060-3, PMID: 39676097 PMC11646999

[21] Jie P Baowen X Honglian M Rui W Xiao H Zhongjun H . Deep learning based on computed tomography predicts response to chemoimmunotherapy in lung squamous cell carcinoma. Aging Dis. (2024). doi: 10.14336/AD.2024.0169, PMID: 38916736 PMC12096918

[22] Jie P Jing Z Dan Z Lushan X Honglian M Xudong Z . Deep learning to estimate durable clinical benefit and prognosis from patients with non-small cell lung cancer treated with PD-1/PD-L1 blockade. Front Immunol. (2022) 13:960459. doi: 10.3389/fimmu.2022.960459, PMID: 36420269 PMC9677530

[23] Zhichao L Guo L Zeping Y Linduo L Xingchen W Jingrong S . Predictive mutation signature of immunotherapy benefits in NSCLC based on machine learning algorithms. Front Immunol. (2022) 13:989275. doi: 10.3389/fimmu.2022.989275, PMID: 36238300 PMC9552174

[24] Seong-Keun Y Conall F Byuri Angela C Bailey GF Catherine YH Elizabeth SK . Prediction of checkpoint inhibitor immunotherapy efficacy for cancer using routine blood tests and clinical data. Nat Med. (2025). doi: 10.1038/s41591-024-03398-5, PMID: 39762425 PMC11922749

[25] Yawei L Wu X Deyu F Yuan L . Informing immunotherapy with multi-omics driven machine learning. NPJ digital Med. (2024) 333:71–4. doi: 10.1038/s41746-024-01043-6, PMID: 38486092 PMC10940614

[26] World medical association declaration of helsinki: ethical principles for medical research involving human participants. Jama. (2024). doi: 10.1001/jama.2024.21972, PMID: 39425955

[27] Ramtin Zargari M . ExplaineR: an R package to explain machine learning models. Bioinf Adv. (2024). doi: 10.1093/bioadv/vbae049, PMID: 38577543 PMC10994716

[28] Gang G Miao Y Wei X Esteban C Yan C . Local activation of p53 in the tumor microenvironment overcomes immune suppression and enhances antitumor immunity. Cancer Res. (2017). doi: 10.1158/0008-5472.CAN-16-2832, PMID: 28280037 PMC5465961

[29] Xiaonan Z Simin M Yifan Y Dushan D Qicai L Saisai L . A TP53 related immune prognostic model for the prediction of clinical outcomes and therapeutic responses in lung adenocarcinoma. Front Immunol. (2022) 13:876355. doi: 10.3389/fimmu.2022.876355, PMID: 35837383 PMC9275777

[30] Xin C Tianqi L Jianqi W Chen Z Gefei G Cunyi Z . Molecular profiling identifies distinct subtypes across TP53 mutant tumors. JCI Insight. (2022). doi: 10.1172/jci.insight.156485, PMID: 36256461 PMC9746906

[31] Max DW Seth BC Danique EMD Martine HVM Maarten S Iris d . Loss of p53 triggers WNT-dependent systemic inflammation to drive breast cancer metastasis. Nature. (2019). doi: 10.1038/s41586-019-1450-6, PMID: 31367040 PMC6707815

[32] Kristen ES Diane AF Christine CA Zoe LC Amanda LB Wentao Y . T cell immune deficiency rather than chromosome instability predisposes patients with short telomere syndromes to squamous cancers. Cancer Cell. (2023). doi: 10.1016/j.ccell.2023.03.005, PMID: 37037617 PMC10188244

[33] Karen HV Kevin MR . p53 and metabolism. Nat Rev Cancer. (2009). doi: 10.1038/nrc2715, PMID: 19759539

[34] Jia J Junjie G Lina G Kaihua L Jie D Fanshuang Z . CNDP1 overexpression by promoter hypomethylation predicts poor prognosis and immunotherapy response in mucosal melanoma. Cancer Sci. (2025). doi: 10.1111/cas.70062, PMID: 40133062 PMC12127094

[35] Yunfeng Y Qianyu L Guoquan Y Yifei Q Wenyun G Songling L . Targeting KMT5C suppresses lung cancer progression and enhances the efficacy of immunotherapy. Advanced Sci. (2025). doi: 10.1002/advs.202407575, PMID: 40126333 PMC12097080

[36] Jian W Yuke C Shiwei L Wanchang L Xiao Albert Z Yefei L . PP2A inhibition causes synthetic lethality in BRCA2-mutated prostate cancer models via spindle assembly checkpoint reactivation. J Clin Invest. (2023). doi: 10.1172/JCI172137, PMID: 37934606 PMC10760972

[37] Robert MS Chung-Han L Alexander NS Matthew DH Ronglai S Yelena YJ . Tumor mutational load predicts survival after immunotherapy across multiple cancer types. Nat Genet. (2019). doi: 10.1038/s41588-018-0312-8, PMID: 30643254 PMC6365097

[38] Qingmiao S Chen X Yifan Z Xin Y Qingfei C Shuwen J . Notch signaling pathway in cancer: from mechanistic insights to targeted therapies. Signal transduction targeted Ther. (2024). doi: 10.1038/s41392-024-01828-x, PMID: 38797752 PMC11128457

[39] Lu Z Du W Jiao X Wang Y Shi J Shi Y . NOTCH1 mutation and survival analysis of tislelizumab in advanced or metastatic esophageal squamous cell carcinoma: A biomarker analysis from the randomized, phase III, RATIONALE-302 trial. J Clin Oncol Off J Am Soc Clin Oncol. (2025) 43:1898–909. doi: 10.1200/JCO-24-01818, PMID: 40179324 PMC12118624

[40] Nicholas JW Zachary S Kelly LA Laura JB Wilson L Charlotte MP . Loss-of-function mutations in Notch receptors in cutaneous and lung squamous cell carcinoma. Proc Natl Acad Sci United States America. (2011). doi: 10.1073/pnas.1114669108, PMID: 22006338 PMC3203814

[41] Nicolas S Ann Marie E Aaron DT Aleksandar DK Kristian C Andrey S . The mutational landscape of head and neck squamous cell carcinoma. Science. (2011). doi: 10.1126/science.1208130, PMID: 21798893 PMC3415217

[42] Matthew AC Sophie de Carné T Sareena R Davide Z Christopher M Míriam MA . Oncogenic RAS signaling promotes tumor immunoresistance by stabilizing PD-L1 mRNA. Immunity. (2017). doi: 10.1016/j.immuni.2017.11.016, PMID: 29246442 PMC5746170

[43] Chengling L Xingchen L Pengjuan C Haiming X Xin L Sailing Z . Circadian rhythm related genes identified through tumorigenesis and immune infiltration-guided strategies as predictors of prognosis, immunotherapy response, and candidate drugs in skin cutaneous Malignant melanoma. Front Immunol. (2025). 16:1513750. doi: 10.3389/fimmu.2025.1513750, PMID: 40191195 PMC11968383

[44] Liu Y Jiahong J Song Y Yaping X Dongsheng H . Circulating tumor DNA analysis to detect minimal residual disease and predict recurrence in patients with resectable pancreatic cancer. J Clin Oncol. (2020). doi: 10.1200/jco.2020.38.15_suppl.e16799 PMC7406781 32850360

[45] Aaron CT Gillianne L Stephanie PLS Kevin Lee Min C Angela T Boon-Hean O . MA07.06 circulating tumor DNA for monitoring minimal residual disease and early detection of recurrence in early stage lung cancer. J Thorac Oncol. (2021). doi: 10.1016/j.jtho.2021.08.144

[46] Hua C Xinyi L Yixin C Pan-Chyr Y Tanxiao H Lele S . Circulating tumor DNA is capable of monitoring the therapeutic response and resistance in advanced colorectal cancer patients undergoing combined target and chemotherapy. J Clin Oncol. (2020). doi: 10.1200/jco.2020.38.15_suppl.e15524, PMID: 32318348 PMC7154135

[47] Lorenzo G Carolina R Andrew AD Marko V Katherine C Whitney LH . Defining resistance mechanisms to CDK4/6 inhibition in hormone receptor-positive HER2-negative metastatic breast cancer (MBC) through a machine learning approach applied to circulating tumor DNA (ctDNA). J Clin Oncol. (2022). doi: 10.1200/jco.2022.40.16_suppl.3055

[48] Mingjun R Yingcheng W Yunfei L Zhengyang F . Immunotherapy guided by immunohistochemistry PD-L1 testing for patients with NSCLC: A microsimulation model-based effectiveness and cost-effectiveness analysis. BioDrugs: Clin immunotherapeutics biopharmaceuticals Gene Ther. (2023). doi: 10.1007/s40259-023-00628-z, PMID: 37792142

[49] Akhrorbek T Dilmurod T Jiyoun K Wooseong K . Feature selection and machine learning approaches for detecting sarcopenia through predictive modeling. Mathematics. (2024). doi: 10.3390/math13010098

