# Supplementary figures and images for: Development and validation of an interpretable machine learning model for predicting progression-free survival after immunotherapy in patients with non-small cell lung cancer: a multicenter study
Source: Front Immunol. 2025 Dec 19;16:1686260. doi: 10.3389/fimmu.2025.1686260 (PMC12757269; doi:10.3389/fimmu.2025.1686260)

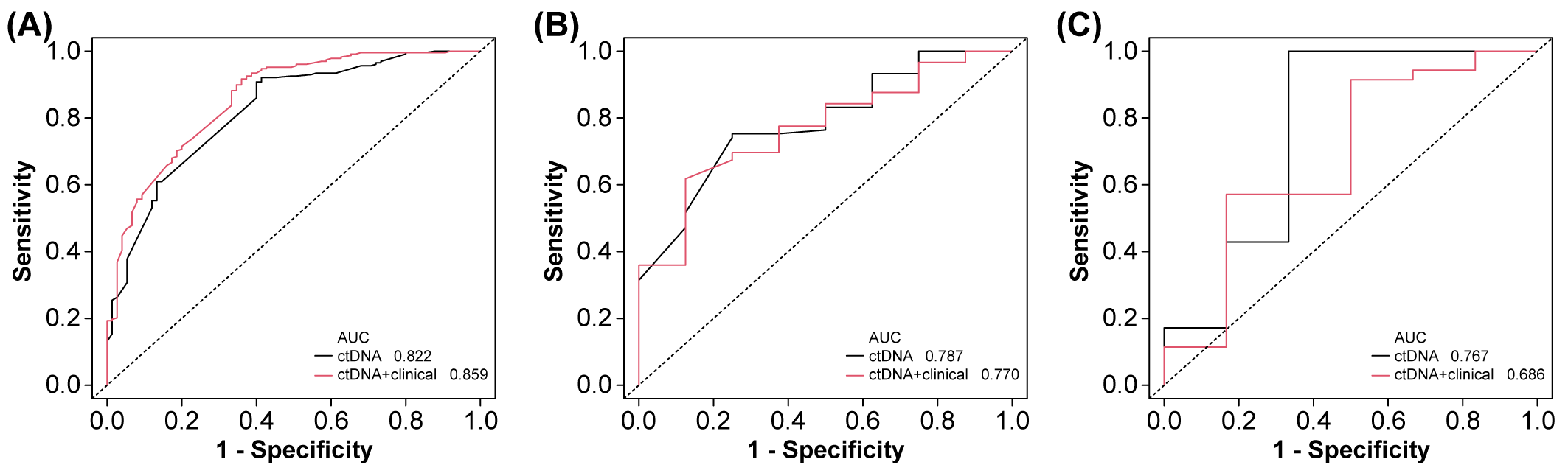

Supplement: Supplementary file 1 [file Image1.tif]

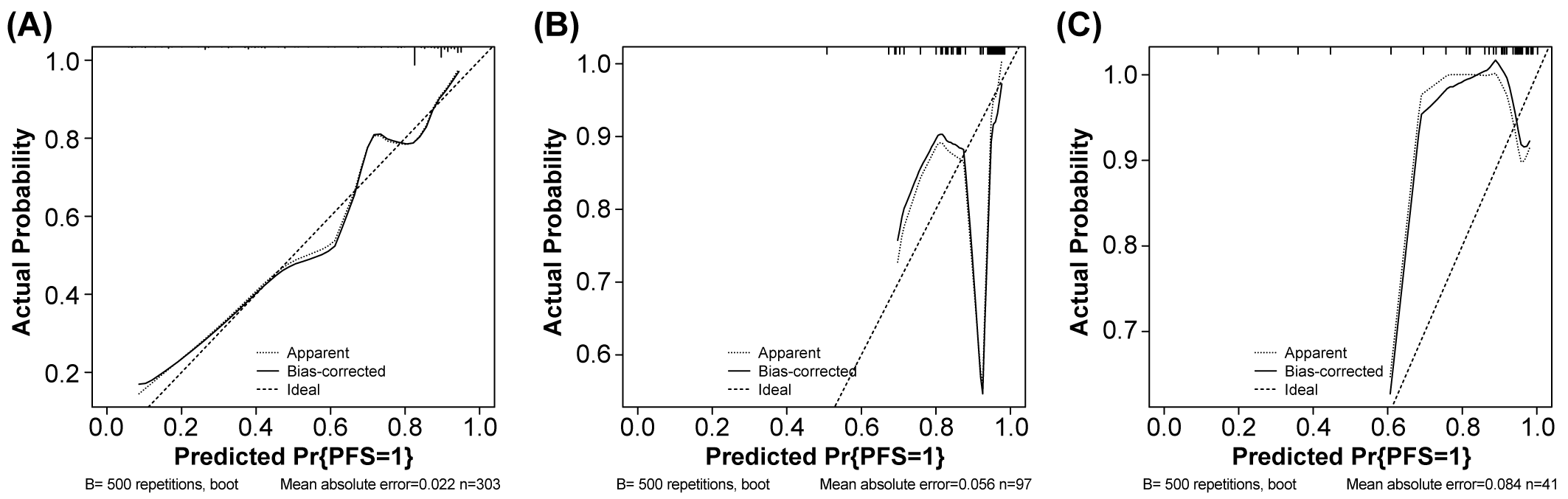

Supplement: Supplementary file 2 [file Image2.tif]
