# Supplementary material for: Development and validation of an interpretable machine learning model for predicting progression-free survival after immunotherapy in patients with non-small cell lung cancer: a multicenter study
Source: Front Immunol. 2025 Dec 19;16:1686260. doi: 10.3389/fimmu.2025.1686260 (PMC12757269; doi:10.3389/fimmu.2025.1686260)
Supplement: Supplementary file 3 [file Table1.docx]

Supplementary Material

# Supplementary Figures and Tables

## Supplementary Figures

**Supplementary Figure 1.** Comparative performance of ctDNA-based vs integrative models across datasets.(**A**) Training set ROC: ctDNA alone vs ctDNA+clinical. (**B**) Validation set ROC: ctDNA alone vs ctDNA+clinical. (**C**) Test set ROC: ctDNA alone vs ctDNA+clinical.

**Supplementary Figure 2.** Assessment of prediction calibration across datasets. (**A**) Training set calibration curve. (**B**) Validation set calibration curve. (**C**) Test set calibration curve.

## Supplementary Tables

**Supplementary table 1 Univariate and Multivariate Cox Regression Analysis of PFS.**

| Variable | Univariate Analysis | | Multivariate Analysis | |
| --- | --- | --- | --- | --- |
|  | HR | *P*-value | HR | *P*-value |
| ABL1 | 0.75 | 0.88 |  |  |
| AKT3 | 51.22 | 0.01 | 5.04 | 0.01 |
| ALK | 0.49 | 0.59 |  |  |
| ALOX12B | 0.24 | 0.4 |  |  |
| APC | 1.37 | 0.66 |  |  |
| ARID1A | 0.58 | 0.62 |  |  |
| ARID1B | 1.24 | 0.84 |  |  |
| ARID2 | 0.72 | 0.52 |  |  |
| ASXL1 | 1.08 | 0.91 |  |  |
| ATM | 0.24 | 0.04 | 0.24 | <0.01 |
| ATR | 1.5 | 0.67 |  |  |
| AXIN1 | 0.27 | 0.47 |  |  |
| AXL | 0.01 | 0.02 | 0.08 | <0.01 |
| BAP1 | 1.04 | 0.96 |  |  |
| BARD1 | 1.17 | 0.84 |  |  |
| BLM | 0.01 | 0.16 | 0.26 | 0.12 |
| BRAF | 1.04 | 0.97 |  |  |
| BRCA1 | 1.12 | 0.89 |  |  |
| BRCA2 | 0.07 | 0.05 | 0.2 | <0.01 |
| BRD4 | 1.28 | 0.85 |  |  |
| BRIP1 | 0.1 | 0.38 |  |  |
| CARD11 | 0.77 | 0.86 |  |  |
| CASP8 | 0.72 | 0.89 |  |  |
| CBL | 5.89 | 0.14 | 2.1 | 0.06 |
| CCND1 | 3.39 | 0.79 |  |  |
| CCND2 | 0.01 | 0.19 | 0.04 | <0.01 |
| CCNE1 | 0.88 | 0.97 |  |  |
| CD274 | 4.53 | 0.72 |  |  |
| CDC73 | 0.2 | 0.75 |  |  |
| CDH1 | 58.85 | 0.07 | 9.32 | 0.02 |
| CDK8 | 0.75 | 0.92 |  |  |
| CDKN2A | 0.1 | 0.06 | 0.18 | <0.01 |
| CHEK2 | 0.48 | 0.29 |  |  |
| CIC | 1.03 | 0.98 |  |  |
| CREBBP | 3.1 | 0.17 | 0.66 | 0.38 |
| CSF1R | 1.68 | 0.78 |  |  |
| CTCF | 17.57 | 0.04 | 5.53 | 0.01 |
| CTNNB1 | 6.45 | 0.27 |  |  |
| CUL3 | 18.83 | 0.1 | 2.98 | 0.06 |
| DDR2 | 1.5 | 0.76 |  |  |
| DICER1 | 0.11 | 0.2 |  |  |
| DIS3 | 1.72 | 0.83 |  |  |
| DNMT3A | 0.72 | 0.37 |  |  |
| DOT1L | 3.29 | 0.35 |  |  |
| EGFR | 0.4 | 0.09 | 0.93 | 0.79 |
| EP300 | 0.23 | 0.12 | 0.48 | 0.04 |
| EPHA3 | 1.31 | 0.78 |  |  |
| EPHA5 | 10.95 | 0 | 3.67 | <0.01 |
| EPHA7 | 1.46 | 0.68 |  |  |
| EPHB1 | 1.54 | 0.66 |  |  |
| ERBB2 | 238.12 | 0 | 12.5 | <0.01 |
| ERBB3 | 0.15 | 0.07 | 0.56 | 0.18 |
| ERBB4 | 1.25 | 0.78 |  |  |
| ERCC2 | 0.04 | 0.6 |  |  |
| ERCC4 | 5.32 | 0.01 | 4.91 | <0.01 |
| ERG | 0.37 | 0.36 |  |  |
| ESR1 | 0.49 | 0.51 |  |  |
| EZH2 | 0.04 | 0.04 | 0.18 | 0.05 |
| FANCC | 0.24 | 0.63 |  |  |
| FAT1 | 1.88 | 0.27 |  |  |
| FBXW7 | 1.61 | 0.68 |  |  |
| FGF19 | 215.42 | 0.02 | 12.49 | <0.01 |
| FGF3 | 1.34 | 0.9 |  |  |
| FGFR1 | 30.55 | 0.07 | 14.65 | <0.01 |
| FGFR2 | 1.12 | 0.9 |  |  |
| FGFR3 | 0.07 | 0.04 | 0.22 | 0.02 |
| FGFR4 | 2.75 | 0.18 | 2.93 | 0.02 |
| FH | 1.24 | 0.9 |  |  |
| FLT1 | 0.52 | 0.67 |  |  |
| FLT3 | 17.68 | 0.02 | 3.41 | <0.01 |
| FLT4 | 0.75 | 0.8 |  |  |
| FOXL2 | 0.14 | 0.25 |  |  |
| FOXP1 | 0.85 | 0.91 |  |  |
| FUBP1 | 7.2 | 0.28 |  |  |
| GATA2 | 3.42 | 0.54 |  |  |
| GATA3 | 13.53 | 0.02 | 4.68 | 0.01 |
| GLI1 | 0.08 | 0.07 | 0.13 | <0.01 |
| GRIN2A | 0.64 | 0.56 |  |  |
| GSK3B | 0.99 | 0.99 |  |  |
| HGF | 0.14 | 0.13 | 0.28 | <0.01 |
| HNF1A | 0.01 | 0.08 | 0.05 | <0.01 |
| HOXB13 | 0.84 | 0.9 |  |  |
| IGF1R | 0.4 | 0.66 |  |  |
| IGF2 | 0.01 | 0.02 | 0.12 | 0.03 |
| IKZF1 | 1.82 | 0.59 |  |  |
| IL7R | 0.13 | 0.02 | 0.34 | 0.04 |
| INHBA | 7.35 | 0.08 | 2.46 | 0.1 |
| INPP4B | 0.6 | 0.65 |  |  |
| IRF4 | 0.27 | 0.45 |  |  |
| IRS2 | 2.51 | 0.49 |  |  |
| JAK1 | 0.56 | 0.83 |  |  |
| JAK2 | 0.06 | 0.21 |  |  |
| JAK3 | 0.29 | 0.42 |  |  |
| KDM5A | 0.35 | 0.34 |  |  |
| KDM5C | 1.57 | 0.81 |  |  |
| KDM6A | 0.46 | 0.6 |  |  |
| KDR | 2.4 | 0.45 |  |  |
| KEAP1 | 3.79 | 0.01 | 4.57 | <0.01 |
| KIT | 18.09 | 0.02 | 1.91 | 0.3 |
| KRAS | 5.63 | 0.01 | 2.28 | 0.01 |
| MAP2K2 | 177.28 | 0.05 | 12.02 | 0.04 |
| MAP3K1 | 2.58 | 0.72 |  |  |
| MAP3K13 | 1.39 | 0.9 |  |  |
| MCL1 | 0.75 | 0.93 |  |  |
| MDM4 | 0.38 | 0.49 |  |  |
| MEF2B | 1.61 | 0.84 |  |  |
| MET | 0.22 | 0.43 |  |  |
| MITF | 0.58 | 0.84 |  |  |
| MPL | 18.52 | 0.06 | 4.43 | 0.02 |
| MSH2 | 0.03 | 0.2 |  |  |
| MSH6 | 0.04 | 0.1 | 0.15 | 0.01 |
| MST1R | 0.42 | 0.5 |  |  |
| MTOR | 0.36 | 0.36 |  |  |
| MYC | 1.28 | 0.87 |  |  |
| MYCN | 29.56 | 0.05 | 4.14 | 0.03 |
| NBN | 3.59 | 0.29 |  |  |
| NCOR1 | 0.25 | 0.14 | 0.26 | 0.02 |
| NF1 | 3.26 | 0.01 | 1.96 | 0.01 |
| NF2 | 1.21 | 0.93 |  |  |
| NFE2L2 | 0.89 | 0.91 |  |  |
| NOTCH1 | 0.27 | 0.08 | 0.33 | 0.01 |
| NOTCH2 | 0.81 | 0.83 |  |  |
| NOTCH3 | 0.51 | 0.49 |  |  |
| NRAS | 111.18 | 0.05 | 5.87 | 0.02 |
| NSD1 | 0.01 | 0.06 | 0.16 | 0.01 |
| NTRK1 | 1.4 | 0.88 |  |  |
| NTRK2 | 0.08 | 0.19 | 0.07 | <0.01 |
| NTRK3 | 0.76 | 0.78 |  |  |
| NUP93 | 2.94 | 0.51 |  |  |
| PALB2 | 0.41 | 0.33 |  |  |
| PARP1 | 0.06 | 0.18 | 0.12 | <0.01 |
| PAX5 | 16.32 | 0.22 |  |  |
| PBRM1 | 0.53 | 0.76 |  |  |
| PDGFRA | 2.8 | 0.27 |  |  |
| PDGFRB | 0.01 | 0.08 | 0.31 | 0.05 |
| PIK3C2G | 0.04 | 0 | 0.11 | <0.01 |
| PIK3C3 | 2.23 | 0.46 |  |  |
| PIK3CA | 2.86 | 0.16 | 3.18 | <0.01 |
| PIK3CB | 11.2 | 0.28 |  |  |
| PIK3CG | 13.89 | 0 | 6.42 | <0.01 |
| PIK3R1 | 161.23 | 0 | 37.78 | <0.01 |
| PLCG2 | 0.05 | 0.05 | 0.31 | 0.02 |
| PMS2 | 7.85 | 0.04 | 2.9 | 0.17 |
| POLD1 | 0.31 | 0.42 |  |  |
| POLE | 0.57 | 0.34 |  |  |
| PRDM1 | 0.12 | 0.39 |  |  |
| PREX2 | 2.84 | 0.21 |  |  |
| PTCH1 | 3.55 | 0.01 | 3.1 | <0.01 |
| PTEN | 39.38 | 0.01 | 5.72 | <0.01 |
| PTPN11 | 17.58 | 0.47 |  |  |
| PTPRD | 0.28 | 0.2 |  |  |
| RAD50 | 3.78 | 0.07 | 1.14 | 0.81 |
| RAD51C | 8.22 | 0.03 | 3.25 | 0.16 |
| RAD54L | 10.55 | 0.05 | 2.46 | 0.2 |
| RAF1 | 6.65 | 0.48 |  |  |
| RARA | 0.75 | 0.85 |  |  |
| RB1 | 10.54 | 0 | 4.55 | <0.01 |
| RBM10 | 0.37 | 0.34 |  |  |
| RET | 43.25 | 0.02 | 4.57 | 0.04 |
| RICTOR | 1.65 | 0.57 |  |  |
| RNF43 | 0.74 | 0.81 |  |  |
| ROS1 | 3.3 | 0.16 | 0.96 | 0.93 |
| RPTOR | 0 | 0.02 | 0 | <0.01 |
| RUNX1 | 0.06 | 0.06 | 0.35 | 0.17 |
| SETD2 | 4.61 | 0.13 | 1.85 | 0.12 |
| SF3B1 | 7.12 | 0.38 |  |  |
| SMAD3 | 0.11 | 0.34 |  |  |
| SMAD4 | 20.34 | 0 | 6.04 | <0.01 |
| SMARCA4 | 0.47 | 0.47 |  |  |
| SMO | 11.44 | 0.19 | 8.7 | <0.01 |
| SOX9 | 1.76 | 0.18 | 1.47 | 0.16 |
| SPEN | 1.11 | 0.91 |  |  |
| SPOP | 411.53 | 0.03 | 4.98 | 0.13 |
| SRC | 16.72 | 0.19 | 1.56 | 0.55 |
| STAG2 | 0.58 | 0.21 |  |  |
| STAT3 | 1.02 | 0.98 |  |  |
| STK11 | 2.28 | 0.32 |  |  |
| SYK | 0 | 0.01 | 0.08 | 0.01 |
| TBX3 | 0.47 | 0.36 |  |  |
| TEK | 0.4 | 0.75 |  |  |
| TET2 | 1.92 | 0.33 |  |  |
| TGFBR2 | 2.8 | 0.46 |  |  |
| TNFAIP3 | 0.95 | 0.99 |  |  |
| TOP1 | 0.16 | 0.27 |  |  |
| TP53 | 5.75 | 0 | 2.17 | <0.01 |
| TP53BP1 | 10.07 | 0 | 5.39 | <0.01 |
| TSC1 | 0.45 | 0.17 | 0.5 | 0.04 |
| TSC2 | 0.79 | 0.75 |  |  |
| TSHR | 0.19 | 0.03 | 0.54 | 0.16 |
| U2AF1 | 108.21 | 0.06 | 2.15 | 0.51 |
| WT1 | 22.76 | 0.01 | 6.63 | <0.01 |
| XPO1 | 0.03 | 0.06 | 0.42 | 0.21 |

**Supplementary table 2 Predictive performance of the XGBoost model and baseline models on the test set.**

| Statistical value | XGBoost | LR | SVM |
| --- | --- | --- | --- |
| Accuracy | 0.95 | 0.90 | 0.85 |
| Precision | 1.00 | 0.90 | 0.95 |
| AUC | 0.77 | 0.69 | 0.75 |
